# Unlocking the gut-liver axis: microbial contributions to the pathogenesis of metabolic-associated fatty liver disease

**DOI:** 10.3389/fmicb.2025.1577724

**Published:** 2025-04-25

**Authors:** Mykhailo Buchynskyi, Iryna Kamyshna, Iryna Halabitska, Pavlo Petakh, Oksana Kunduzova, Valentyn Oksenych, Oleksandr Kamyshnyi

**Affiliations:** ^1^Department of Microbiology, Virology, and Immunology, I. Horbachevsky Ternopil National Medical University, Ternopil, Ukraine; ^2^Department of Medical Rehabilitation, I. Horbachevsky Ternopil National Medical University, Ternopil, Ukraine; ^3^Department of Therapy and Family Medicine, I. Horbachevsky Ternopil National Medical University, Ternopil, Ukraine; ^4^Department of Biochemistry and Pharmacology, Uzhhorod National University, Uzhhorod, Ukraine; ^5^Institute of Metabolic and Cardiovascular Diseases (I2MC), National Institute of Health and Medical Research (INSERM) 1297, Toulouse III University, Toulouse, France; ^6^Department of Clinical Science, University of Bergen, Bergen, Norway

**Keywords:** MAFLD, microbiota, FXR, TGR5, metabolism

## Abstract

Metabolic dysfunction-associated fatty liver disease (MAFLD) is a complex metabolic disorder characterized by hepatic lipid accumulation and subsequent inflammation. This condition is closely linked to metabolic syndrome and obesity, with its prevalence rising due to sedentary lifestyles and high-calorie diets. The pathogenesis of MAFLD involves multiple factors, including insulin resistance, lipotoxicity, oxidative stress, and inflammatory responses. The gut microbiota plays a crucial role in MAFLD development, with dysbiosis contributing to liver inflammation through various mechanisms, such as enhanced intestinal permeability and the translocation of bacterial products like lipopolysaccharide (LPS). Microbial metabolites, including short-chain fatty acids (SCFAs) and bile acids, influence hepatic function and immune responses, with potential implications for disease progression. Specific gut microbiome signatures have been identified in MAFLD patients, offering potential diagnostic and therapeutic targets. Moreover, gut-derived toxins, such as endotoxins, lipopolysaccharides, trimethylamine-N-oxide and bacterial metabolites, significantly influence liver damage and inflammation, highlighting the complex interplay between the gut microbiome and hepatic health. This review comprehensively examines the complex interplay between the gut microbiota and MAFLD, focusing on underlying pathogenic mechanisms, potential biomarkers, and emerging microbiome-targeted therapeutic strategies for disease management.

## 1 Introduction

Metabolic-associated fatty liver disease (MAFLD) represents a significant global health burden, affecting an estimated 25–30% of the adult population worldwide (Younossi et al., 2019). This complex metabolic disorder, previously termed non-alcoholic fatty liver disease (NAFLD), has emerged as the most prevalent liver disease (Fouad et al., 2020). The escalating incidence of MAFLD is attributed to a constellation of factors, including sedentary lifestyles, diminished physical activity, and dietary patterns characterized by caloric intake substantially exceeding energy expenditure (Guo et al., 2022).

The pathogenesis of MAFLD is intricately linked to the regulatory mechanisms underlying metabolic syndrome and obesity (Yki-Järvinen, 2014). The hallmark feature of MAFLD is hepatic lipid accumulation, resulting in lipotoxicity. This lipotoxic state may progress to metabolic-associated steatohepatitis (MASH) (Tilg et al., 2021). Without intervention, MASH can lead to fibrosis, potentially culminating in cirrhosis and hepatocellular carcinoma (Huang et al., 2022).

High-fat diet (HFD) consumption has been implicated in MAFLD development (Velázquez et al., 2019). HFD-induced lipotoxicity significantly contributes to hepatic insulin resistance, a pivotal factor in the pathogenesis of both type-2 diabetes mellitus (T2DM) and MAFLD (Birkenfeld and Shulman, 2014). The mechanisms involved in MAFLD development and its progression to MASH interact at multiple levels, forming a complex network of processes conceptualized as the “multiple hit” hypothesis (Tilg et al., 2021).

The “multiple hit” hypothesis posits that MASH progression results from a complex interplay of numerous pathophysiological factors (Tilg et al., 2021). These factors encompass genetic predisposition, insulin resistance, aberrant lipid metabolism, mitochondrial dysfunction, and lipotoxicity. Additionally, oxidative stress, endoplasmic reticulum (ER) stress, ethanol consumption, and compromised gut barrier integrity contribute to the disease process. Furthermore, gut-derived endotoxins, particularly LPS, and dysregulation of cytokine and adipokine profiles play crucial roles in MAFLD pathogenesis (Tilg et al., 2021; Liu et al., 2016).

Alterations in gut microbial composition, or dysbiosis, are implicated in the augmentation of intestinal permeability, resulting in the translocation of bacterial components, including LPS and peptidoglycans, as well as other microbial metabolites, into the portal circulation. This process initiates hepatic inflammatory cascades through the Toll-like receptor 4 (TLR4)/nuclear factor kappa B (NF-κB) signaling pathway (Tilg et al., 2021; Liu et al., 2016). Furthermore, SCFAs, specifically butyrate, acetate, and propionate, have been demonstrated to modulate insulin sensitivity, lipid metabolism, and inflammatory responses, thereby possessing the potential to either exacerbate or attenuate disease progression (Harte et al., 2010; Leung et al., 2016). Concurrently, secondary BAs, which are subject to microbial modification within the gut lumen, interact with the farnesoid X receptor (FXR) and the Takeda G-protein-coupled receptor 5 (TGR5), exerting influence over hepatic lipid homeostasis and inflammatory processes (Mori et al., 2022; Buchynskyi et al., 2024b).

The human microbiota, a diverse ecosystem of microorganisms colonizing various anatomical niches, has emerged as a critical factor in MAFLD pathophysiology. While these microorganisms inhabit multiple body sites, the gastrointestinal tract harbors the most substantial and diverse microbial population, hence the prevalent use of the term “gut microbiota” (Vallianou et al., 2021). In adult humans, the gut bacterial population is predominantly composed of two phyla: the Gram-positive Firmicutes and the Gram-negative Bacteroidetes (Vallianou et al., 2019). Microorganisms, through their involvement in diverse metabolic processes, modulate host health by conferring protection against pathogens and influencing innate immune system development. Perturbations in the homeostatic balance of these microbial communities, termed dysbiosis, are associated with disease states (Vallianou et al., 2019).

Alterations in the composition and function of the gut microbiota, termed dysbiosis, have been implicated in various hepatic pathologies, including MAFLD. The pathogenic potential of gut microbiota dysbiosis in MAFLD is mediated through multiple mechanisms. Intestinal bacteria and their metabolic byproducts can translocate to the liver via the portal venous system, directly influencing hepatic pathophysiology (Wiest et al., 2017; Nawrot et al., 2021). Moreover, gut microbiota-derived metabolites, including SCFAs, BAs, LPS, choline, and trimethylamine (TMA), have demonstrated correlations with MAFLD severity and fibrosis stage. These findings suggest the potential utility of microbial metabolites as non-invasive diagnostic and prognostic biomarkers for MAFLD (Loomba et al., 2019).

The intestinal barrier, a complex anatomical and functional entity, serves as a critical interface mediating gut-liver interactions. This barrier functions to restrict the systemic dissemination of microbes and their potentially harmful products while facilitating the selective absorption of nutrients into the portal circulation for hepatic processing (Rochoń et al., 2024). Maintenance of gut-liver axis homeostasis is contingent upon the intricate regulation of microbial communities. The liver plays a pivotal role in this regulatory process through bidirectional communication with the gut microbiota, influencing microbial composition and metabolic activity (Albillos et al., 2020).

Recent investigations have revealed significant perturbations in gut microbiota composition and gut-liver axis function in human subjects with MAFLD (Yang et al., 2023). These alterations appear to be intricately involved in the pathomechanism of the disease, potentially contributing to its onset, progression, and associated complications (Yang et al., 2023). The elucidation of these complex interactions between the gut microbiome and MAFLD pathogenesis opens new avenues for research and may lead to the development of innovative diagnostic tools and therapeutic interventions.

In conclusion, the multifaceted nature of MAFLD pathogenesis, encompassing metabolic dysregulation, gut microbiota dysbiosis, and altered gut-liver communication, necessitates a comprehensive and integrative approach to understanding and managing this increasingly prevalent liver disease.

## 2 Pathogenesis of MAFLD

The pathogenesis of MAFLD is characterized by a complex interplay of multiple factors, encompassing genetic predisposition, environmental influences, and lifestyle determinants factors (Sookoian and Pirola, 2023). At the core of MAFLD’s pathophysiology lies insulin resistance, a metabolic aberration that precipitates an augmented release of fatty acids from adipose tissue (Parthasarathy et al., 2020).

The process of lipid accumulation in MAFLD involves a cascade of cellular events. As fat absorption escalates, hypertrophic adipocytes stimulate the proliferation of hyperplastic adipocytes. This cellular expansion attracts an influx of macrophages, which subsequently release pro-inflammatory mediators known as adipokines. Over time, these hypertrophic adipocytes undergo functional deterioration, becoming highly lipolytic. This dysfunction results in the excessive production of free fatty acids (FFA), further exacerbating insulin resistance. The surplus of FFA leads to ectopic lipid accumulation, surpassing the hepatic capacity for fatty acid oxidation and intracellular storage (Elkanawati et al., 2024).

The hepatic uptake of these excess fatty acids culminates in hepatic steatosis, marking the initial phase of MAFLD (Zhang et al., 2018). The progression from simple steatosis to steatohepatitis involves a multifaceted process characterized by oxidative stress, mitochondrial dysfunction, and endoplasmic reticulum stress.

The accumulation of FFAs within the liver disrupts hepatic lipid homeostasis, leading to elevated levels of hepatic triglycerides, free cholesterol, and other lipid metabolites. This lipotoxic state within hepatocytes triggers a cascade of deleterious consequences, including mitochondrial dysfunction and oxidative stress. The impaired mitochondrial function, coupled with the excessive influx of FFAs, overwhelms the electron transport chain, leading to an increased production of reactive oxygen species (ROS). This heightened oxidative stress further exacerbates cellular damage and promotes inflammation (Tzeng and Lee, 2024).

Adding to this intricate interplay of pathological mechanisms is the compromise of the intestinal barrier, often observed in the context of MAFLD (Chopyk and Grakoui, 2020). Circulating lipid metabolites can disrupt the integrity of tight junctions between intestinal epithelial cells, increasing intestinal permeability (Chopyk and Grakoui, 2020). This breach allows for the translocation of bacterial products, such as LPS, from the gut lumen into the portal circulation. LPS, a potent immunostimulatory molecule, activates TLR4 signaling in hepatic Kupffer cells and other immune cells (Rivera et al., 2007), leading to the production of pro-inflammatory cytokines, such as TNF-α and IL-6 (De Muynck et al., 2021).

Inflammation, a hallmark of MAFLD progression, is orchestrated by the activation of the innate and adaptive immune systems. Pro-inflammatory cytokines, released by activated immune cells in response to lipotoxicity and bacterial products, play a central role in mediating hepatocellular injury, promoting fibrogenesis, and driving the progression from simple steatosis to steatohepatitis (De Muynck et al., 2021).

The intricate interplay of these pathophysiological processes highlights the complexity of MAFLD pathogenesis. It is not merely an excess of hepatic fat accumulation but rather a complex metabolic disorder characterized by a web of interconnected mechanisms. Understanding these interconnected pathways is critical for developing effective therapeutic strategies to combat this increasingly prevalent disease.

## 3 The importance of the intestine microbiota—liver communication

The intricate communication between the intestinal microbiota and the liver represents a critical axis in maintaining systemic homeostasis and metabolic health. This bidirectional interaction is mediated through a complex network of anatomical and physiological components, each playing a crucial role in the delicate balance between host and microbiota.

The gut barrier, a sophisticated multi-layered defense system, serves as the primary interface between the host and the luminal environment. This barrier comprises a mucus layer, strategically organized into two distinct strata: an outer, more permeable layer and an inner, densely packed layer (Paradis et al., 2021). Beneath this mucus fortress lie the epithelial cells, which form a secondary, equally critical line of defense. These epithelial cells are intricately connected by tight junctions (TJs), molecular complexes that regulate paracellular permeability with exquisite precision (Paradis et al., 2021).

Tight junctions, by virtue of their molecular architecture and functional plasticity, perform a dual role of paramount importance. They act as selective gatekeepers, impeding the invasion of the intestinal epithelial cells by potentially pathogenic microorganisms while simultaneously facilitating the controlled absorption of essential nutrients (Benedé-Ubieto et al., 2024). This discriminatory function is fundamental to maintaining the delicate balance between nutrient acquisition and host defense (Benedé-Ubieto et al., 2024).

The immune system plays an equally pivotal role in this complex interplay between the gut and liver. IgA, secreted locally by plasma cells residing in the lamina propria, serves as a first line of immunological defense (Vallianou et al., 2024). These antibodies bind and neutralize invading microorganisms, preventing their adherence to the epithelial surface and subsequent translocation. In parallel, the release of IL-23 by antigen-presenting cells triggers the activation of group 3 innate lymphoid cells. These cells, in turn, produce IL-22, a cytokine crucial for maintaining epithelial barrier integrity and promoting antimicrobial peptide production (Vallianou et al., 2024).

The vascular and lymphoid components of the gut-liver axis further reinforce this defensive network. The anatomical arrangement of the portal circulation, where the majority of small and large intestinal blood flow converges before reaching the liver, creates a unique immunological niche (Paradis et al., 2021). As blood from the intestines reaches the hepatic sinusoids, it encounters a specialized population of endothelial cells. These cells play a crucial role in activating Kupffer cells, the liver’s resident macrophages (Paradis et al., 2021). Upon activation, Kupffer cells strategically relocate to the periportal area, where they form an additional immunological barrier. This strategic positioning allows Kupffer cells to efficiently intercept and neutralize pathogens and gut-derived toxins that may have breached the intestinal barrier (Paradis et al., 2021).

The gut-liver defensive mechanism operates bidirectionally, with a significant liver-to-gut component complementing the gut-to-liver axis. This hepatic contribution to intestinal defense is primarily mediated through bile, a complex mixture of BAs, IgA, antimicrobial peptides, and bicarbonates. This hepatic secretion exhibits potent host-defending properties, forming a chemical barrier against potential pathogens in the intestinal lumen (Mori et al., 2022). Bile acids, the predominant component of bile, exert their antibacterial effects through multiple mechanisms. Directly, their detergent-like properties can disrupt bacterial cell membranes, leading to cell lysis. Indirectly, bile acids activate specific receptors, notably the FXR and TGR5, which modulate various aspects of host metabolism and immunity (Mori et al., 2022; Buchynskyi et al., 2024b).

**Figure 1:**
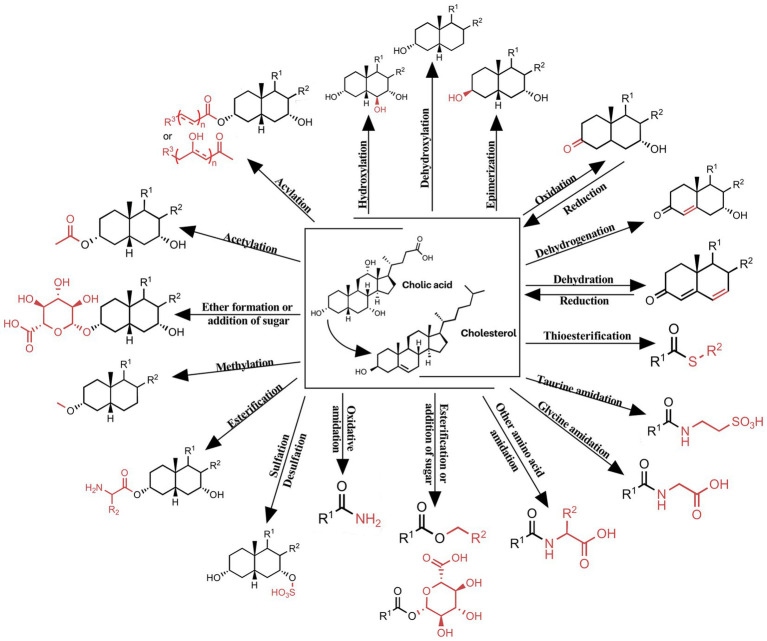
Chemical modifications of bile acids observed in humans. Shows the modifications of the bile acid core: hydroxylation, dehydroxylation, epimerization, oxidation, dehydrogenation, dehydration; modifications of the carboxy: thioesterification, taurine amidation, glycine amidation, esterification or addition of sugar, oxidative amidation and other amino acid amidation; modifications of the hydroxyl: sulfation, esterification, methylation, acetylation, acylation, ether formation or addition of sugar.

Bile acids, a diverse group of steroid acids, are synthesized in the liver from cholesterol through a complex series of enzymatic reactions. Following their synthesis, these molecules are transported to the intestine, where they undergo extensive metabolic transformations mediated by the gut microbiota. These microbial-dependent modifications include deconjugation, dehydroxylation, oxidation, epimerization, and re-conjugation, among other reactions (Buchynskyi et al., 2023c) (Figure 1).

This intricate interplay between host-derived bile acids and microbial metabolism results in a diverse pool of bile acid species with varying physiological effects.

Bile acids are conventionally categorized into primary and secondary bile acids, based on their origin and chemical structure. Primary BAs, predominantly cholic acid (CA) and chenodeoxycholic acid (CDCA), are synthesized *de novo* in hepatocytes and subsequently excreted into the bile duct (Fiorucci and Distrutti, 2019). In contrast, secondary SBAs, including lithocholic acid (LCA), deoxycholic acid (DCA), ursodeoxycholic (UDCA), and their respective isoforms (such as isolithocholic acid), are products of microbial transformation of primary bile acids in the small intestine (Fiorucci and Distrutti, 2019).

**Figure 2:**
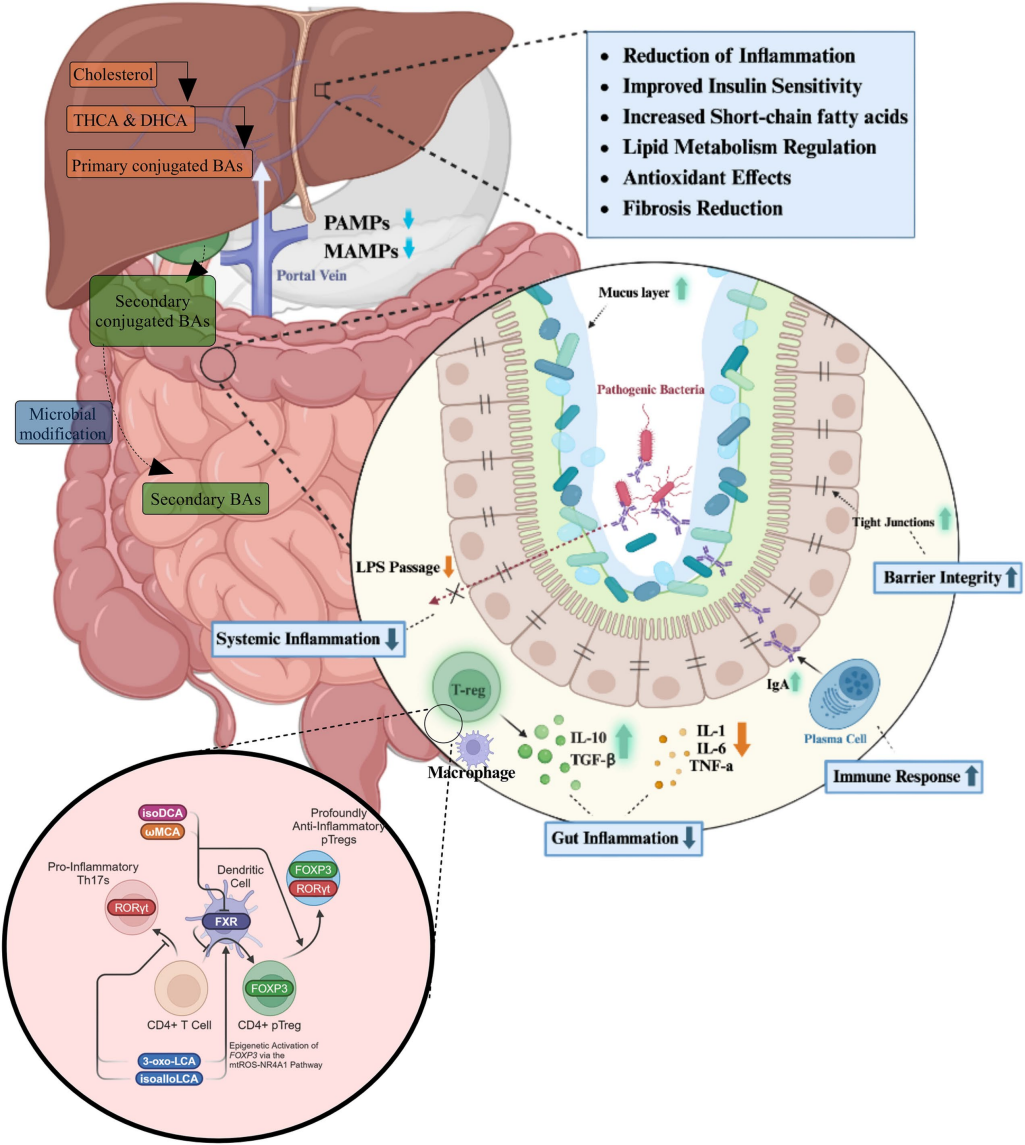
The roles of bile acids in gastrointestinal homeostasis and inflammation reduction. Bile acids, synthesized in the liver from cholesterol, are secreted into the intestine as primary conjugated bile acids, where they undergo microbial modifications to form secondary bile acids. These bile acids play a crucial role in maintaining gut homeostasis by modulating microbial composition, enhancing gut barrier integrity, and influencing immune cell activity. Within the intestinal environment, bile acids regulate the balance between pro-inflammatory and anti-inflammatory responses. They promote the secretion of IgA, strengthen tight junctions, and reduce gut inflammation through the modulation of macrophages and regulatory T cells (T-regs), which secrete anti-inflammatory cytokines such as IL-10 and transforming growth factor-beta (TGF-β). Concurrently, they suppress the production of pro-inflammatory cytokines, including IL-1, IL-6, and tumor necrosis factor-alpha (TNF-α). At a systemic level, bile acids influence immune function by interacting with nuclear receptors such as the FXR, which modulates CD4^+^ T cell differentiation. The activation of FXR supports the expansion of anti-inflammatory regulatory T cells expressing FOXP3 while suppressing the differentiation of pro-inflammatory Th17 cells expressing RORγt. This immunomodulatory effect contributes to a reduction in systemic inflammation by decreasing lipopolysaccharide (LPS) translocation into the bloodstream. The physiological outcomes of these processes include improved insulin sensitivity, enhanced lipid metabolism, increased short-chain fatty acid production, and antioxidant effects.

The physiological roles of bile acids extend far beyond their classical function in lipid emulsification and absorption (Figure 2).

Primary bile acids play a crucial role in maintaining intestinal microbiota homeostasis through direct inhibition of pathogenic bacterial overgrowth. Moreover, they act as endogenous agonists for the FXR in the intestinal mucosa (Fiorucci and Distrutti, 2019). The potency of FXR activation follows the order: CDCA > DCA > LCA > CA, while UDCA acts as an FXR inhibitor. FXR activation triggers the expression of downstream defense genes in the ileal mucosa, conferring protection to intestinal epithelial cells against bacterial and microbial degradation (Fiorucci and Distrutti, 2019).

Furthermore, FXR activation exerts profound effects on hepatic metabolism. It downregulates the expression of liver X receptor (LXR) and sterol regulatory element-binding protein 1c (SREBP-1c), leading to a reduction in fatty acid and triglyceride synthesis in the liver (Han et al., 2019). This mechanism contributes to the attenuation of steatogenesis and gluconeogenesis. Concurrently, FXR upregulates hepatic glycogen synthesis through the activation of fibroblast growth factor (FGF) 15/19, peroxisome proliferator-activated receptor gamma (PPARγ), glucose transporter type 4 (GLUT-4), and glucagon-like peptide-1 (GLP-1), collectively improving insulin sensitivity (Han et al., 2019).

**Figure 3:**
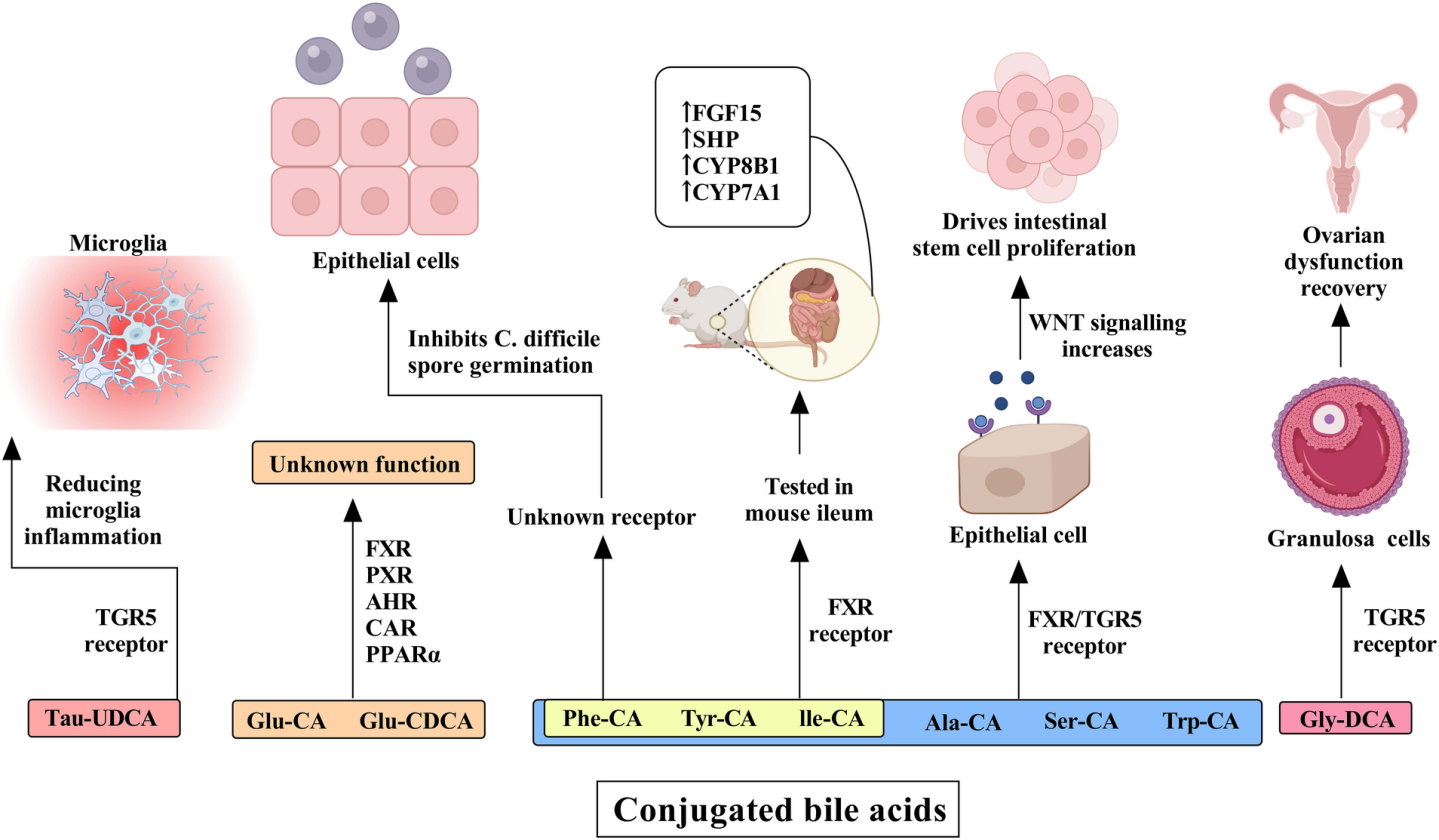
The influence of conjugated bile acids on receptors and their effects on human cells and tissues. Tau-ursodeoxycholic acid (Tau-UDCA) interacts with the TGR5 receptor, resulting in a reduction in microglial inflammation and underscoring its potential role in neuroinflammatory regulation. Glucuronidated bile acids, including glucuronidated cholic acid (Glu-CA) and glucuronidated chenodeoxycholic acid (Glu-CDCA), engage multiple nuclear receptors such as FXR, PXR, AHR, CAR, and PPARα, although their precise physiological functions remain unclear. Further modifications, including the conjugation of alanine (Ala-CA), serine (Ser-CA), and tryptophan (Trp-CA), as well as phenylalanine-conjugated cholic acid (Phe-CA), tyrosine-conjugated cholic acid (Tyr-CA), and isoleucine-conjugated cholic acid (Ile-CA), influence epithelial cells through FXR and TGR5 receptor activation, thereby enhancing WNT signaling and promoting intestinal stem cell proliferation. The latter three bile acid conjugates have been tested in the mouse ileum and have been shown to activate the FXR receptor, leading to the increased expression of FGF15, SHP, CYP8B1, and CYP7A1. Additionally, glycine-deoxycholic acid (Gly-DCA) interacts with the TGR5 receptor in granulosa cells, facilitating ovarian dysfunction recovery.

**Figure 4:**
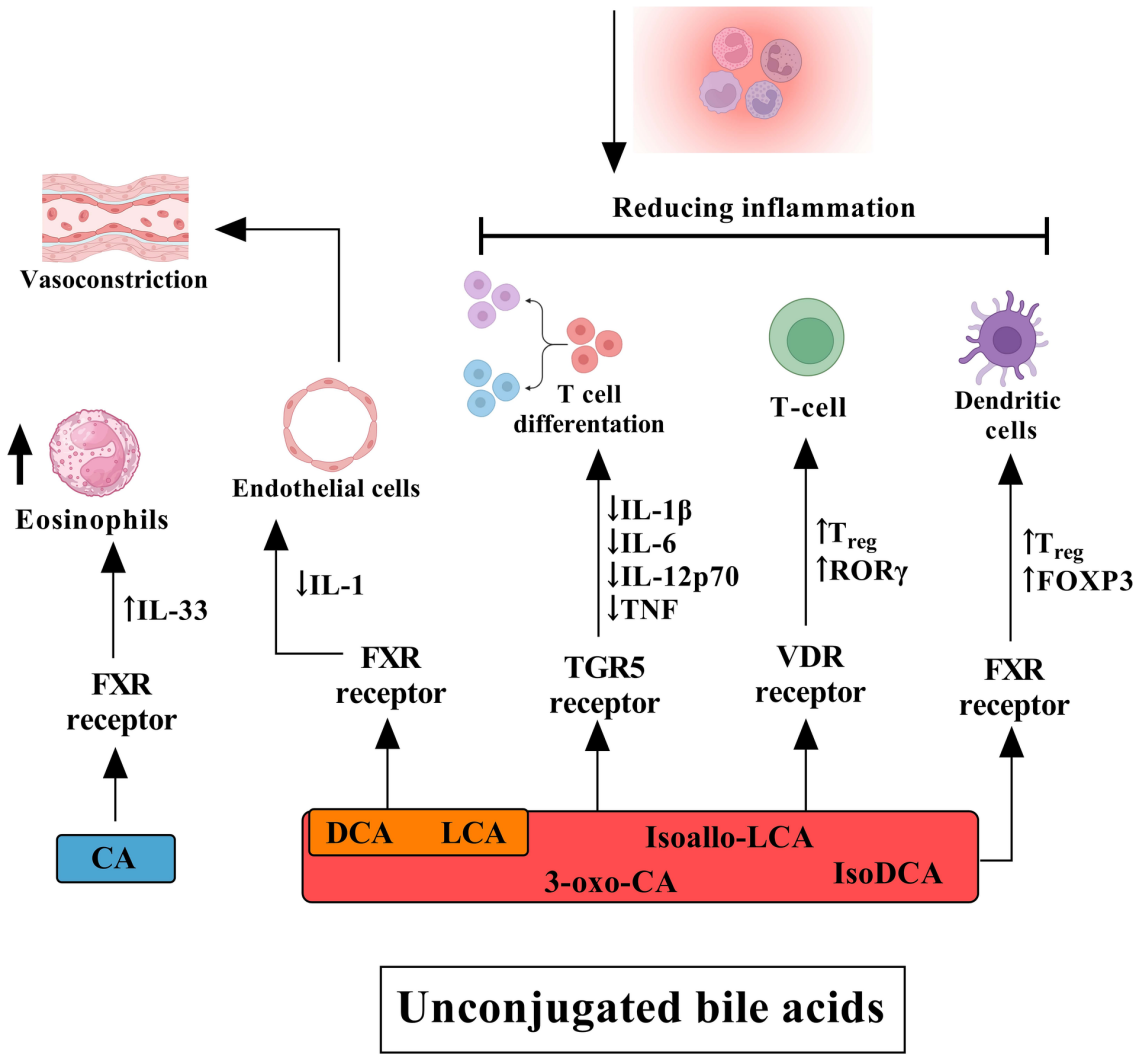
The influence of unconjugated bile acids on receptors and their effects on human cells and tissues. Cholic acid primarily activates the FXR in eosinophils, inducing the release of interleukin (IL)-33. DCA and lithocholic acid (LCA) also act through FXR in endothelial cells, stimulating the secretion of IL-1, which contributes to a cascade of inflammatory events, including vasoconstriction. DCA, LCA, isoallo-LCA, isoDCA, and 3-oxo-CA target the TGR5 on T cells. Activation of TGR5 suppresses the production of pro-inflammatory cytokines such as IL-1β, IL-6, IL-12p70, and TNF while promoting T cell differentiation. Furthermore, these bile acids facilitate the differentiation of T cells into regulatory T cells (Tregs) through activation of the vitamin D receptor (VDR), leading to increased expression of FOXP3 and RORγt. Additionally, FXR signaling in dendritic cells plays a crucial role in Treg modulation and the broader anti-inflammatory response mediated by conjugated bile acids. Collectively, these mechanisms contribute to the reduction of inflammation.

The G protein-coupled bile acid receptor 1 (GPBAR1), also known as TGR5, represents another classical bile acid receptor. TGR5 is preferentially activated by secondary bile acids, with the potency order being LCA > DCA > CDCA > CA. The physiological balance of secondary bile acids is critical; insufficient levels lead to reduced FXR activity and increased systemic inflammation, while excessive amounts can induce cellular DNA damage through the generation of ROS (Fang et al., 2022). TGR5 activation in intestinal L cells promotes the expression of GLP-1, which enhances insulin synthesis and secretion, thereby protecting islet β-cells from apoptosis and improving glucose homeostasis (Maczewsky et al., 2019). Bile acids also exert other influences, which are illustrated in Figures 3, 4.

Sayin et al. (2013) elucidated a novel aspect of the microbiota-bile acid interaction, demonstrating that the gut microbiota not only metabolizes bile acids but also modulates signaling through the FXR. Their groundbreaking research revealed that the microbiota plays a crucial role in deconjugating tauro-beta-muricholic acid (TβMCA), a naturally occurring FXR antagonist in mice. This deconjugation process effectively promotes FXR signaling, highlighting the intricate interplay between microbial metabolism and host nuclear receptor activation (Sayin et al., 2013). Furthermore, their findings underscored the importance of the microbiota in the production of secondary bile acids, which serve as potent ligands for FXR (Kuipers et al., 2014).

In a related study, it was observed that microbiota-induced adipose tissue inflammation and increased hepatic expression of genes involved in lipid uptake occurred in an FXR-dependent manner (Parséus et al., 2017). This observation further emphasizes the complex relationship between the gut microbiome, bile acid metabolism, and host metabolic regulation. Intriguingly, when the altered gut microbiota from high-fat fed FXR-deficient mice was transferred into germ-free (GF) mice, it resulted in less weight gain compared to the microbiota transferred from wild-type (WT) counterparts (Sayin et al., 2013). This finding suggests that the absence of FXR signaling in the donor mice led to alterations in the gut microbiome that conferred a degree of protection against diet-induced obesity in the recipient GF mice (Parséus et al., 2017).

The reciprocal relationship between bile acids and the gut microbiota is further exemplified by the ability of bile acids to shape the microbial community composition (Wahlström et al., 2016). Bile acids promote the growth of bile acid-metabolizing bacteria while inhibiting the proliferation of bile-sensitive bacterial species. This selective pressure exerted by bile acids plays a crucial role in maintaining microbial homeostasis in the gut. Studies have demonstrated that biliary obstruction, which impedes the flow of bile into the intestine, leads to bacterial overgrowth and translocation in the small intestine. Remarkably, this pathological state can be reversed through the administration of bile acids (Wahlström et al., 2016), underscoring the critical role of bile acids in regulating microbial ecology and intestinal barrier function.

The antimicrobial effects of bile acids are multifaceted. Directly, they exert their bactericidal action by disrupting bacterial cell membranes, a consequence of their detergent-like properties. Indirectly, bile acids modulate the immune system through FXR-mediated mechanisms, inducing the transcription of antimicrobial agents such as inducible nitric oxide synthase (iNOS) and IL-18 (Inagaki et al., 2006). These FXR-induced factors contribute to the host’s defense against pathogenic microorganisms, further illustrating the complex interplay between bile acids, nuclear receptors, and the immune system in maintaining gut homeostasis (Inagaki et al., 2006).

Both FXR and TGR5 receptors play pivotal roles in regulating bile acid synthesis through a negative feedback loop. This regulation is achieved by inhibiting the expression of cholesterol 7 alpha-hydroxylase (CYP7A1), the rate-limiting enzyme in bile acid synthesis (Goodwin et al., 2000). In patients with MAFLD, a reduced expression of FXR has been observed, coinciding with elevated levels of serum triglycerides (TGs) (Yang et al., 2010). Moreover, these patients exhibit an altered bile acid profile, characterized by an elevated ratio of DCA to CDCA (Jiao et al., 2018). These perturbations in bile acid levels and compositions may result in a diminished capacity of the FXR and TGR5 receptors to exert their regulatory functions, potentially contributing to the development of insulin resistance and exacerbating lipid accumulation in the liver (Puri et al., 2018).

## 4 Gut microbiota and hepatic immune function

The intricate relationship between the gut microbiota and hepatic immune function is mediated by a complex array of microbial metabolites, with LPS and SCFAs playing particularly pivotal roles (Harte et al., 2010; Leung et al., 2016). These microbial-derived compounds exert profound and multifaceted effects on liver physiology, demonstrating both beneficial and detrimental impacts contingent upon their relative abundance and the overall metabolic milieu.

Lipopolysaccharide, a key structural component of gram-negative bacterial cell walls, has emerged as a critical mediator in the pathogenesis of various liver disorders (Harte et al., 2010). Concurrently, short-chain fatty acids, primarily comprising butyrate, propionate, and acetate, have been identified as crucial signaling molecules that modulate hepatic function (Leung et al., 2016). The delicate balance between these microbial metabolites significantly influences liver homeostasis, with perturbations in their levels potentially contributing to the onset and progression of hepatic pathologies (Furet et al., 2010).

It is noteworthy that these microbial-derived metabolites extend their influence beyond hepatic function, playing substantial roles in systemic metabolic regulation. Their involvement in obesity and metabolic disturbances, including T2DM, has been extensively documented (Cani et al., 2007). Murine models have provided compelling evidence demonstrating elevated circulating LPS levels during obesity-induced metabolic dysfunction. This increase in LPS activates insulin resistance signaling cascades across multiple tissue types, underscoring the systemic impact of this gut-derived endotoxin (Cani et al., 2007).

Short-chain fatty acids, despite their well-established beneficial effects on metabolic health, present a paradoxical aspect in energy homeostasis. These microbial fermentation products contribute significantly to host energy metabolism and, under certain circumstances, may promote weight gain (Canfora et al., 2015). This dichotomous nature of SCFAs is further exemplified by clinical observations reporting elevated fecal SCFA concentrations in obese individuals compared to their lean counterparts (Schwiertz et al., 2010).

The role of dietary choline in the interplay between gut microbiota and liver function has garnered significant attention in recent years. Choline-deficient diets have long been established as a robust experimental model for inducing MAFLD, precipitating both hepatic steatosis and marked alterations in gut microbial composition (Raubenheimer et al., 2006; Yu et al., 2014). Choline, an essential nutrient serving as a precursor to phosphatidylcholine and acetylcholine, can undergo oxidation to form betaine. Clinical investigations have revealed a diminished betaine-to-choline ratio in patients presenting with more severe manifestations of MAFLD, suggesting a potential link between choline metabolism and disease progression (Dumas et al., 2006).

The metabolic fate of dietary choline is intricately linked to gut microbial activity. Specific gut bacteria metabolize choline to TMA, which is subsequently oxidized to trimethylamine N-oxide (TMAO) by hepatic flavin-containing monooxygenase 3 (FMO3) (Wang et al., 2011). Metagenomic analyses have associated elevated TMAO levels with individuals harboring the Prevotella enterotype, with certain microbial taxa exhibiting increased abundance in subjects displaying higher TMAO concentrations (Koeth et al., 2013). Animal studies have demonstrated a positive correlation between MAFLD severity and elevated urinary TMA and TMAO levels (Dumas et al., 2006). Furthermore, independent clinical investigations have established a significant association between increased TMAO levels and MAFLD severity when compared to healthy control subjects (Chen et al., 2016).

The gut microbiota serves as a crucial mediator in bile acid metabolism, orchestrating the biotransformation of primary bile acids into their secondary counterparts through a series of intricate enzymatic processes. This microbial-mediated conversion is not merely a metabolic footnote but a fundamental process that profoundly influences host physiology (Di Ciaula et al., 2017). Bile acids, once considered solely as facilitators of lipid emulsification and absorption, are now recognized as potent signaling molecules that regulate a diverse array of physiological processes, including cholesterol homeostasis, glucose metabolism, and energy expenditure (Di Ciaula et al., 2017).

Emerging evidence from both human and animal studies has elucidated the complex relationship between microbial bile acid biotransformation and the pathogenesis of MAFLD and its progression to MASH. The microbial enzymes responsible for bile acid modifications, including bile salt hydrolases (BSH) for deconjugation, and 7α-dehydroxylases for dehydroxylation, play pivotal roles in shaping the bile acid pool. These enzymatic activities not only alter the physicochemical properties of bile acids but also modulate their signaling capacity through nuclear receptors such as FXR and TGR5 (Ridlon et al., 2014; Arab et al., 2017).

The production of TMAO, a gut microbiota-derived metabolite, introduces an additional layer of complexity to the intricate relationship between microbial metabolism and hepatic function. TMAO has been demonstrated to exert a profound influence on bile acid homeostasis through the inhibition of key enzymes in bile acid synthesis, notably CYP7A1 and sterol 27-hydroxylase (CYP27A1). This inhibitory action results in a quantitative reduction of the bile acid pool, potentially altering the delicate balance of lipid metabolism and inflammatory processes in the liver (Chen et al., 2016).

The intricate interplay between gut microbiota, bile acid metabolism, and liver function is further exemplified in patients with advanced cirrhosis (Aron-Wisnewsky et al., 2020). These individuals exhibit profound alterations in both bile acid conversion pathways and microbiota composition. Specifically, cirrhotic patients demonstrate a marked proliferation of Enterobacteriaceae, concomitant with a significant depletion of beneficial bacterial families such as Lachnospiraceae, Ruminococcaceae, and the genus Blautia (Aron-Wisnewsky et al., 2020).

A study conducted in 2019 revealed a correlation between elevated levels of 3-(4-hydroxyphenyl) lactate and increased severity of MAFLD fibrosis in two independent cohorts, totaling 312 participants (Caussy et al., 2018). This metabolite is a gut microbiota-derived product of aromatic amino acid metabolism. These findings align with another study demonstrating reduced microbial gene richness and alterations in aromatic and branched-chain amino acid metabolism in patients with varying degrees of steatosis (Hoyles et al., 2018).

Gut microbiota-derived ethanol may also contribute to MAFLD pathophysiology. Studies have shown that children with MAFLD harbor an increased abundance of ethanol-producing bacteria compared to obese or healthy controls (Zhu et al., 2013). Additionally, adults with MASH exhibit elevated breath ethanol concentrations in the absence of alcohol consumption (Zhu et al., 2013), suggesting increased gut microbiota-derived ethanol production in this population. Escherichia enriched in MAFLD patients is capable of ethanol synthesis, inducing oxidative stress that is involved in MAFLD progression (Zhu et al., 2013).

Research employing inflammasome-deficient murine models indicates that gut microbiome modulation plays a pivotal role in hepatic health, specifically influencing the progression of MAFLD (Henao-Mejia et al., 2012). However, in the absence of direct causal evidence, the alternative hypothesis, wherein alterations in gut microbial composition are a consequence rather than a cause of liver pathology, cannot be discounted. Specifically, the potential for hepatic steatosis to induce an increased abundance of Escherichia within the gut warrants consideration. This scenario, however, appears improbable. Spencer et al. (2011) demonstrated that the induction of fatty liver disease resulted in a reduction in the abundance of *Proteobacteria*, the phylum to which *Escherichia* belongs, within the gut microbiome.

In addition to *Escherichia*, alternative gut microbial genera, such as *Bacteroides* (Frantz and McCallum, 1979), *Bifidobacterium* (Amaretti et al., 2007), and *Clostridium* (Weimer and Zeikus, 1977), have been identified as capable of endogenous ethanol production. The cumulative ethanol generated by these diverse microbial populations may impose a significant burden on hepatic ethanol metabolism. Consequently, an elevated prevalence of ethanol-producing *Escherichia* within the gut microbiome may constitute a pathogenic factor in the transition from obesity to MASH (Bashiardes et al., 2016).

Research has identified *Klebsiella pneumoniae* as a bacterium capable of producing ethanol from glucose, even in the absence of alcohol intake, supporting this hypothesis (Yuan et al., 2019).

SCFAs, comprising butyrate, acetate, and propionate, are primarily produced in the colon through microbial fermentation of indigestible complex carbohydrates (dietary fiber) (Furet et al., 2010; Kolodziejczyk et al., 2019). Proposed as key contributors to hepatic triglyceride accumulation and weight gain (Samuel et al., 2008), SCFAs are implicated in fatty acid synthesis and gluconeogenesis (den Besten et al., 2013). Comparative studies of MAFLD, MASH, and healthy controls have revealed elevated fecal SCFA concentrations in MAFLD and/or MASH patients (Rau et al., 2018) concurrent with increased abundance of SCFA-producing bacterial groups.

The physiological impact of SCFAs is multifaceted and context-dependent. While SCFAs can exert beneficial metabolic effects, they also possess anti-inflammatory properties. Research has demonstrated that activation of G protein-coupled receptor 43 (GPR43) by SCFAs results in the attenuation of proinflammatory responses and reduction of T cell infiltration (Sun et al., 2017).

Given these complex interactions, elucidating the intricate interplay between LPS, bile acid metabolism, and SCFAs in the development and progression of MAFLD remains a critical area of investigation. Further research in this domain may yield valuable insights into the pathogenesis of MAFLD and potentially inform novel therapeutic strategies.

## 5 Microbiome signature in MAFLD patients

The dysfunction of the gut-liver axis, precipitated by intestinal bacterial overgrowth, dysbiosis, and compromised intestinal permeability, exerts a profound influence on the pathogenesis and progression of MAFLD (Rinaldi et al., 2021).

Interventional studies have demonstrated that modulation of the gut microbiota through probiotic administration results in amelioration of hepatic injuries, normalization of metabolic parameters, and attenuation of inflammatory chemokine levels in MAFLD patients (Ma et al., 2013).

Miele et al. (2009) initially demonstrated a correlation between MAFLD in humans and elevated intestinal permeability, attributing this phenomenon to the heightened incidence of small intestinal bacterial overgrowth (SIBO) within this patient population. The observed increase in permeability is posited to result from the disruption of intercellular tight junctions, thereby potentially assuming a significant role in the pathogenic mechanisms underlying MAFLD. Conversely, Loguercio et al. (2005) have reported that probiotic interventions can mitigate hepatic injury associated with MAFLD and enhance liver functionality. This therapeutic effect is thought to be mediated through multiple mechanisms, including the inhibition of pathogenic bacterial proliferation, reduction of SIBO, restoration of gastrointestinal barrier integrity, and modulation of the immune response (Macfarlane and Cummings, 2002; Guarner and Malagelada, 2003), collectively contributing to the amelioration of MAFLD.

Research has established that probiotics can augment epithelial barrier function (Malasanos and Stacpoole, 1991), concurrently mitigating intestinal permeability and endotoxemia in individuals with hepatic pathologies (Malaguarnera et al., 2010). Moreover, probiotics exert influence over host metabolic processes through diverse mechanisms, including the regulation of energy extraction from dietary substrates and the modulation of genes implicated in substrate metabolism (Vanni and Bugianesi, 2009).

**Figure 5:**
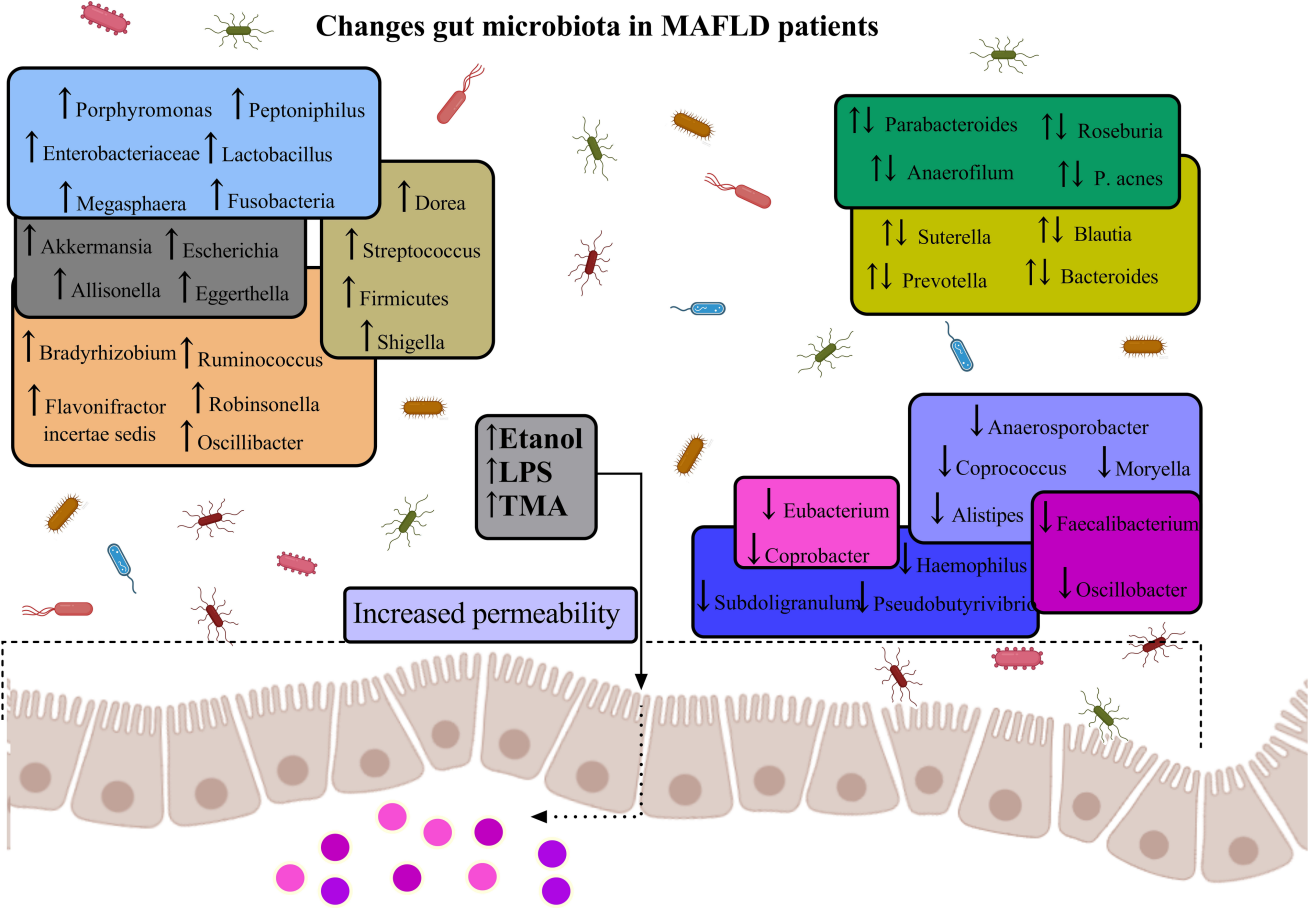
Gut microbiota diversity in MAFLD. The figure illustrates the dysregulation of specific intestinal microbiota, which may contribute to the elevation of inflammatory factors via their metabolic byproducts. Intestinal dysbiosis can induce inflammatory responses in both the intestinal and hepatic tissues, a process potentially mediated by the translocation of endotoxins and bacteria consequent to increased intestinal permeability. This phenomenon results in an augmented risk of both localized and systemic low-grade inflammation, coupled with a diminished anti-inflammatory capacity within the intestine, thereby exacerbating the progression of MAFLD (Aron-Wisnewsky et al., 2020; Wang et al., 2016; Shen et al., 2017; Forlano et al., 2022; Carpi et al., 2022; Loomba et al., 2017).

Recent investigations have revealed the potential utility of gut microbiome signatures as non-invasive diagnostic biomarkers in MAFLD and cirrhosis (Aron-Wisnewsky et al., 2020). Several studies have reported a reduced diversity of the gut microbiota in MAFLD patients compared to healthy individuals (Aron-Wisnewsky et al., 2020; Wang et al., 2016; Shen et al., 2017; Forlano et al., 2022; Carpi et al., 2022; Loomba et al., 2017) (Figure 5). A comprehensive meta-analysis of MAFLD patients unveiled specific alterations in gut microbial composition, characterized by increased abundance of *Escherichia*, *Prevotella*, and *Streptococcus* genera, concomitant with decreased populations of *Coprococcus*, *Faecalibacterium*, and *Ruminococcus* in fecal samples (Li et al., 2021).

The expansion of the *Proteobacteria* phylum, particularly *Escherichia coli* and members of the *Enterobacteriaceae* family, has been associated with increased intestinal permeability and elevated portal lipopolysaccharide levels. These changes may trigger inflammasome activation, contributing to hepatic injury (DuPont and DuPont, 2011). *Prevotella*, a bacterial genus linked to diets rich in fruits and vegetables, has been implicated in SCFA production (De Filippis et al., 2019). The meta-analysis revealed a consistent increase in *Prevotella* abundance across MAFLD studies.

Conversely, bacterial taxa that exhibited reduced abundance in MAFLD patients, such as *Ruminococcaceae* and *Faecalibacterium*, are known to produce SCFAs through fermentation of dietary soluble fibers. These SCFAs activate free fatty acid receptors (FFARs), including G-protein coupled receptor 43 and 41 (GPR43, GPR41) (Zhang et al., 2015). This activation pathway inhibits proinflammatory functions in neutrophils, monocytes, and macrophages, thereby reducing the production of TNF-*α* and monocyte chemotactic protein-1. The efficacy of SCFA-rich diets in alleviating insulin resistance and inflammation has been demonstrated in both experimental mouse models and clinical trials (Kimura et al., 2020). Consequently, the decreased abundance of *Ruminococcaceae* and *Faecalibacterium* may result in lower SCFA levels in the gut, potentially exacerbating the inflammatory processes implicated in MAFLD pathogenesis.

A comprehensive study conducted by Yang et al. (2023) identified specific microbial taxa associated with MAFLD patients. Their findings revealed that *Ruminococcus obeum* and *Alistipes* were significantly enriched in healthy individuals compared to MAFLD patients. *Alistipes*, a member of the *Rikenellaceae* family, has been consistently observed to be depleted in MAFLD patients (Zhu et al., 2013). Moreover, *Alistipes* has demonstrated notable anti-inflammatory properties in both human and animal studies. Several investigations have correlated the presence of *Alistipes* genus with a healthy metabolic state (Mancabelli et al., 2017).

Conversely, the same study reported an enrichment of *Dorea*, *Lactobacillus*, and *Megasphaera* genera in the MAFLD cohort (Mancabelli et al., 2017). While *Dorea* is generally considered a component of the healthy gut microflora, its increased abundance has been associated with inflammatory conditions such as inflammatory bowel disease (IBD), suggesting a potential pro-inflammatory role for this bacterium (Shahi et al., 2017). Research by Rocha-Ramírez et al. (2017) has demonstrated that *Lactobacillus* species can stimulate TNF-α production, indicating that the enrichment of *Lactobacillus* in MAFLD patients may contribute to alterations in inflammatory factors implicated in disease progression.

Comparative analyses of gut microbial composition have revealed that MAFLD patients exhibit lower proportions of *Bacteroidetes* and higher proportions of *Prevotella* and *Porphyromonas* species relative to healthy controls. In patients with MASH, an increased abundance of ethanol-producing bacteria has been observed in their gut microbiome, accompanied by elevated blood ethanol concentrations. These findings suggest a potential role for alcohol-producing microbiota in MASH pathogenesis (Zhu et al., 2013).

A focused investigation of MASH patients demonstrated an increased relative abundance of *Bacteroidetes* compared to non-MASH subjects. Furthermore, the proportion of *Prevotella* was significantly diminished in patients with MASH and advanced fibrosis (*F* ≥ 2) compared to those with mild fibrosis (F0/1). Metagenomic profile analysis associated the presence of advanced fibrosis (*F* ≥ 2) with alterations in carbohydrate, lipid, and amino acid metabolism pathways (Boursier et al., 2016).

A noteworthy study by Del Chierico et al. (2017) employed integrated metagenomic and metabolomic approaches to elucidate the microbial and metabolic signatures of MAFLD. Their findings corroborated the inverse relationship between microbial diversity and obesity/MAFLD severity when compared to healthy controls. Importantly, they identified *Oscillospira* as a potential marker of hepatic health, while noting increased abundances of *Ruminococcus* and *Dorea* correlating with MAFLD progression toward MASH (Del Chierico et al., 2017). Of particular interest, the researchers proposed that a combination of low *Oscillospira* abundance and elevated 2-butanone levels may serve as a specific intestinal signature profile for pediatric MAFLD. These bacterial strains and metabolites differ from those identified by Loomba et al. (2017), who defined a signature comprising several strains and metabolites associated with the progression from mild/moderate MAFLD to advanced fibrosis in adult populations.

Investigations have indicated that certain bacterial species within the genus *Turicibacter* may exert deleterious physiological effects on the host (Vanni and Bugianesi, 2009; Peña-Rodríguez et al., 2022). Notably, Ran et al. (2024) and Frantz and McCallum (1979) reported a positive correlation between the relative abundance (RA) of and serum alanine aminotransferase (ALT) and aspartate aminotransferase (AST) levels. Furthermore, they demonstrated that therapeutic interventions employing *Bifidobacterium* spp., either alone or in combination with rosuvastatin, resulted in improved hepatic function, potentially mediated through a reduction in *Turicibacter* RA. Additionally, a comparative analysis of MAFLD patients with significant liver fibrosis versus those with no or mild fibrosis revealed elevated RAs of *Turicibacter*, *Sarcina*, *Enterobacter*, and in the former group (Rodriguez-Diaz et al., 2022). These findings suggest a potential role for these bacterial taxa in the progression of MAFLD to advanced liver fibrosis.

While the growing body of evidence substantiates the role of intestinal dysbiosis in MAFLD pathogenesis, the heterogeneity of findings across studies underscores the need for further comprehensive investigations to elucidate the precise mechanisms and establish consistent microbial signatures associated with disease progression.

## 6 Microbe toxin and liver damage

While the role of microbial-derived products in driving advanced liver disease and its complications, such as hepatic encephalopathy, is well-established, the contribution of dysbiosis observed in earlier stages of liver diseases to hepatic inflammation remains ambiguous. The intestinal epithelial barrier, a critical component in host defense against bacterial invasion, has been demonstrated to be compromised in various chronic inflammatory disorders, including MAFLD. The gut microbiome is postulated to play a pivotal role in maintaining the integrity of this epithelial barrier (Chopyk and Grakoui, 2020).

TLRs, the most extensively characterized pattern recognition receptors, are capable of initiating inflammatory responses that contribute to the progression of liver injury. In human subjects, dysbiosis has been associated with alterations in the intestinal barrier, resulting in increased intestinal permeability. This permeability leads to bacterial translocation, followed by the activation of pro-inflammatory pathways upon binding to specific hepatic receptors (Rahman et al., 2016; Pendyala et al., 2012; Peterson and Artis, 2014). TLRs are multiprotein complexes that recognize pathogen-associated molecular patterns (PAMPs), such as bacterial peptidoglycans, LPS, double-stranded DNA and RNA (dsDNA, dsRNA) (Heymann and Tacke, 2016), as well as danger-associated molecular patterns (DAMPs) produced during cellular stress or death (Guo et al., 2015). TLR-mediated intracellular signaling involves the activation of inflammasomes. Upon stimulation by specific TLRs, an intracellular cascade is initiated, culminating in the secretion of biologically active cytokines IL-1β and IL-18, which are implicated in inflammation and cell death (Szabo and Iracheta-Vellve, 2015). Accumulating evidence suggests a role for inflammasomes in MAFLD pathogenesis. However, conflicting results have been reported, with some studies indicating that the absence of inflammasome components may protect against liver injury in experimental MAFLD models, while others have demonstrated that their absence is associated with more aggressive disease progression (Mridha et al., 2017; Stienstra et al., 2011; Henao-Mejia et al., 2012).

Macrophages, key components of the innate immune system, comprise diverse subpopulations in the liver, including resident Kupffer cells and recruited monocyte-derived macrophages (Kolios et al., 2006). These cells regulate hepatic immune homeostasis through phagocytosis and antigen presentation (Krenkel and Tacke, 2017). Traditionally, gut-derived endotoxins, particularly LPS, have been identified as primary factors contributing to macrophage activation. Under physiological conditions, constitutive exposure to LPS educates liver macrophages, fostering LPS tolerance and downregulating TLRs (Wu et al., 2015). Pathologically, mounting evidence suggests a crucial role for excessive activation of liver macrophages, induced by the LPS-TLR axis (specifically TLR4), in MAFLD progression (Rivera et al., 2007). The modulation of this specific axis presents a potentially efficacious therapeutic strategy for MAFLD. Notably, the application of novel bioactive peptides, specifically EWYF and EWFY, has demonstrated a reduction in hepatic steatosis, hepatic injury, and proinflammatory responses in murine models. In silico molecular docking analyses suggest that the observed therapeutic effects of EWYF and EWFY may be attributed to their antagonistic activity against fructokinase and inhibitory effects on TLR4, thereby contributing to the attenuation of MAFLD pathogenesis (Wayal et al., 2025).

The increased susceptibility of hepatic macrophages to LPS may be further mediated by lipids (termed lipotoxicity), leading to enhanced release of pro-inflammatory cytokines (Kawaratani et al., 2008), recruitment of effector immune cells (Leroux et al., 2012), and ROS activation (Kudo et al., 2009). Furthermore, early colonization of the infant gut microbiota originating from obese mothers has been shown to increase susceptibility to MAFLD via impaired phagocytosis of hepatic macrophages (Soderborg et al., 2018). In addition to LPS, certain bacterial metabolites also modulate the immune state of hepatic macrophages. Tryptophan metabolites, including tryptamine and indole-3-acetate (I3A), have been demonstrated to reduce pro-inflammatory cytokine production in macrophages through activation of the aryl hydrocarbon receptor (Krishnan et al., 2018).

Emerging evidence has identified N,N,N-trimethyl-5-aminovaleric acid (TMAVA), a gut microbiota-derived metabolite, as a potential driver of hepatic steatosis in MAFLD. Serum levels of TMAVA have been observed to be elevated in MAFLD patients and have been shown to exacerbate hepatic lipid accumulation (Zhao et al., 2020). This microbial metabolite is produced through the gut microbial conversion of trimethyllysine, highlighting the role of specific bacterial metabolic pathways in the pathogenesis of MAFLD.

Furthermore, the gut microbiome-derived metabolite phenylacetate has been implicated as a direct contributor to hepatic steatosis. Hoyles et al. (2018) demonstrated that fecal transfer from obese women into mice resulted in the development of hepatic steatosis, a phenotype that was recapitulated by feeding phenylacetate to the recipient mice. This finding underscores the potential for gut microbial metabolites to directly influence the development of fatty liver disease.

The role of endotoxin, a key microbiota-derived driver, has been particularly highlighted in the more advanced stages of liver disease. Patients with MASH have been shown to exhibit higher circulating endotoxin levels compared to those with simple steatosis. Moreover, hepatocytes in MASH livers have been found to be positive for endotoxin, accompanied by an increased abundance of TLR4-positive hepatic macrophages (Carpino et al., 2020).

Specific microbial metabolites, such as phenylacetate, have been implicated in hepatic lipid accumulation in obese females, thereby contributing to the pathogenesis of MASH. At the microbial community level, patients with MAFLD exhibited a significant increase in the relative abundance of *Proteobacteria*, *Enterobacteriaceae*, and *Escherichia* spp., when compared to healthy controls (Del Chierico et al., 2017). Similarly, pediatric populations with steatosis or MASH demonstrated a depletion of *Oscillospira* spp., concomitant with an elevation in the abundance of *Dorea* and *Ruminococcus* spp. (Del Chierico et al., 2017). These microbiota alterations associated with metabolic liver disease were correlated with increased concentrations of hepatotoxic molecules, including 2-butanone and 4-methyl-2-pentanone (Del Chierico et al., 2017).

In addition to TLRs and nucleotide-binding oligomerization domain-like receptors (NLRs) have also been implicated in MAFLD pathogenesis. These pattern recognition receptors are expressed by various cell types within the liver and are activated by gut-derived microbial components. The activation of TLRs and NLRs leads to the production of numerous cytokines and chemokines, ultimately driving liver inflammation (Xu et al., 2018).

These studies are important, as they suggest that bacterial components are of importance in various aspects of MAFLD, finally resulting in fibrosis and cirrhosis (Luo et al., 2016; Petrasek et al., 2013; Dela Peña et al., 2005).

## 7 The influence of pharmacological agents on gut microbiota and MAFLD progression

**Figure 6:**
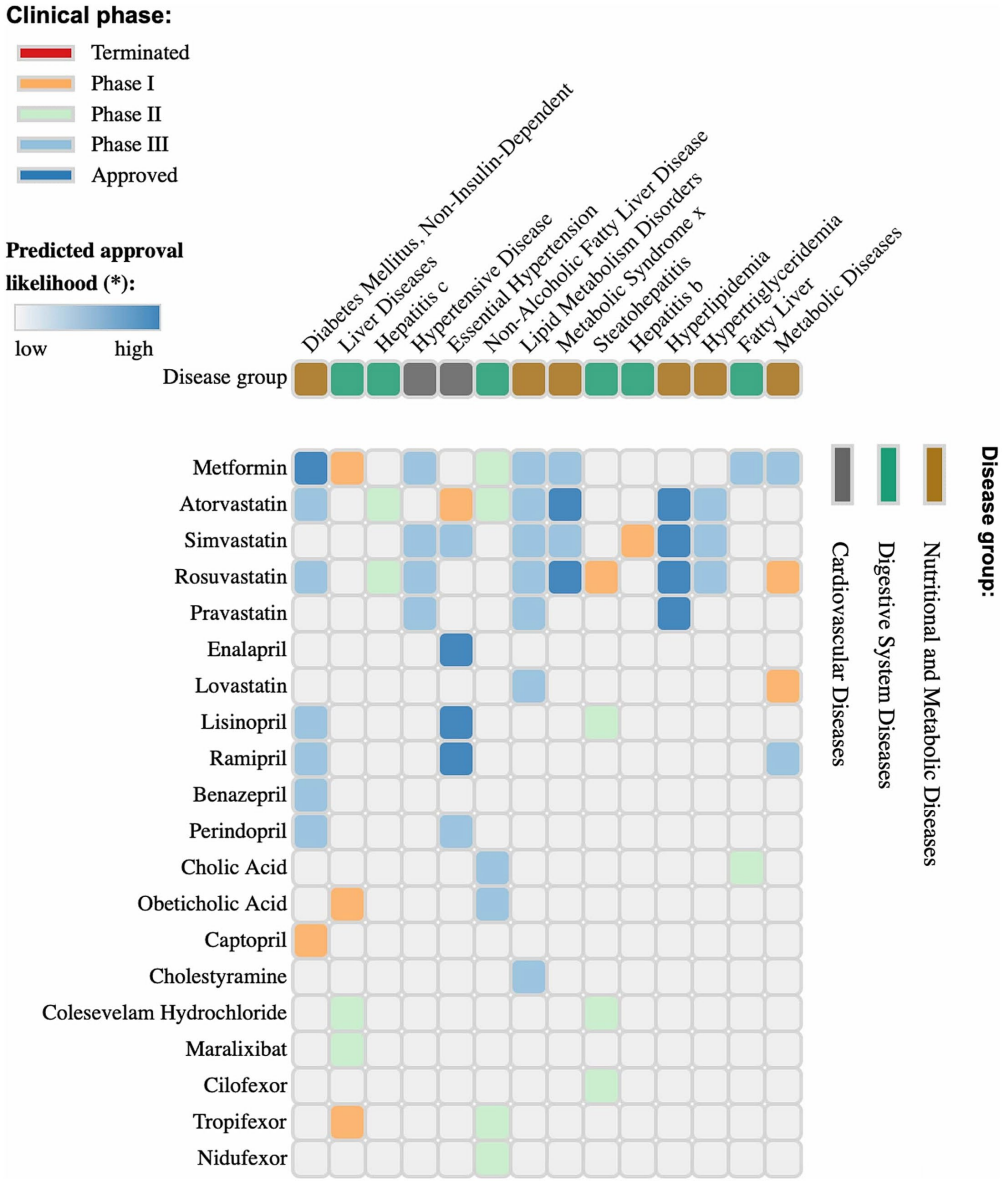
Clinical phases and predicted approval probability of drugs for metabolic, cardiovascular, and digestive system diseases. The image presents a matrix of pharmaceutical agents, their target diseases, and corresponding clinical trial phases. The color coding indicates trial status: terminated (red), phase I (orange), phase II (green), phase III (cyan), and approved drugs (blue). The upper section of the graph categorizes diseases into metabolic, cardiovascular, and digestive system disorders. The intensity of the blue shading in the matrix cells represents the predicted probability of drug approval for a specific condition. This analysis provides valuable insights into the prospects of pharmaceutical agents, their current status in the clinical pipeline, and the likelihood of future approval. Figure 5 was generated using https://repurposedrugs.org, accessed on 15.02.2025.

Pharmacological agents used in treating metabolic and cardiovascular diseases can significantly influence the composition and functional activity of the gut microbiota, which plays a crucial role in the pathogenesis of MAFLD (Figure 6) (Wang et al., 2022; Nesci et al., 2023). The interaction between these drugs and the microbiota occurs through various mechanisms, including alterations in microbial populations, modulation of SCFA production, bile acid metabolism, and regulation of systemic inflammation (Pi et al., 2024; Facchin et al., 2024; Buchynskyi et al., 2023a) (see Table 1).

**Table 1:** Impact of medications on gut microbiota.

Drug name	References	Pharmacological class	Changes in gut microbiota
Metformin	Wu et al. (2017)	Anti-diabetic drug	Increases *Escherichia coli*, *Bifidobacterium*, *Akkermansia muciniphila*, *Shewanella*, and *Blautia*. These changes may contribute to its antidiabetic effects
Atorvastatin and rosuvastatin	Kim et al. (1947), Wilmanski et al. (2022), and Nolan et al. (2017)	Statins	Increase *Bacteroides*, *Butyricimonas*, and *Mucispirillum*. These bacteria are linked to inflammation in the ileum (Zheng et al., 2022). A gut microbiome rich in Bacteroides and low in diversity is associated with stronger statin effects (Pasta et al., 2025). Rosuvastatin also increases *Lachnospiraceae*, *Rikenella*, and *Coprococcus* in HFD mice (Buchynskyi et al., 2023b)
ACE inhibitors	Robles-Vera et al. (2020) and Dong et al. (2022)	Antihypertensive drugs	Reduce harmful bacteria like *Enterobacter* and *Klebsiella* while increasing beneficial ones like *Odoribacter*. In a rat study, losartan improved gut microbiota diversity, restored the *Firmicutes*/*Bacteroidetes* ratio, and enhanced intestinal integrity by increasing tight junction proteins (Huang et al., 2022)
Obeticholic acid	Liu et al. (2023)	FXR agonist	Increases *Akkermansia muciniphila*, *Bifidobacterium*, *Bacteroides*, *Alistipes*, *Lactobacillus*, *Streptococcus thermophilus*, and *Parasutterella excrementihominis* in HFD mice. Alters bile acid metabolism, potentially reducing non-alcoholic fatty liver disease (MAFLD)
UDCA	Pearson et al. (2019)	FXR antagonist	Increases *Faecalibacterium prausnitzii* and reduces *Ruminococcus gnavus*
Gut microbiome modifier			
Probiotic VSL#3	Jena et al. (2020)	Probiotics	Reduces hepatic inflammation, improves insulin sensitivity, and restores gut microbiota balance by increasing *Lachnospiraceae*, *Ruminococcus*, and *Faecalibacterium* while decreasing *Bacteroidaceae*, *Porphyromonadaceae*, and *Helicobacteraceae*
*Lactobacillus bulgaricus* and *Streptococcus thermophilus*	Aller et al. (2011)	Probiotics	Reduce liver enzyme levels (ALT, AST, GGT) but have no effect on anthropometric or cardiovascular parameters
Probiotic yogurt (*Lactobacillus acidophilus* La5, *Bifidobacterium lactis* Bb12)	Nabavi et al. (2014)	Probiotics	Lowers ALT, AST, total cholesterol, and LDL cholesterol without affecting glucose, triglycerides, or HDL cholesterol
Multistrain probiotic supplementation	Sepideh et al. (2016)	Probiotics	Reduces fasting blood sugar, insulin resistance, and IL-6, while TNF-α remains unchanged
Probiotic cocktail	Manzhalii et al. (2017)	Probiotics	Lowers ALT, reduces liver stiffness, BMI, and cholesterol levels. GGT reduction is not significant
Symbiter omega	Kobyliak et al. (2018)	Probiotic with omega-3	Reduces fatty liver index, total cholesterol, triglycerides, and systemic inflammation markers (IL-1β, TNF-α, IL-8, IL-6, IFN-γ)
Probiotic cocktail	Famouri et al. (2017)	Probiotics	Decreases ALT, AST, cholesterol, LDL-C, triglycerides, and waist circumference. Improves liver ultrasound results in a significant proportion of patients

Fecal microbiota transplantation		
Study type	Patients	Outcomes
LFMT-capsules vs. Placebo (NCT05821010)	MAFLD	Recruitment status
FMT via nasojejunal tube (NCT02469272)	Diabetes and MAFLD	Improved HOMA index
FMT via duodenal infusion (NCT02721264)	MAFLD and MASH	Lowered liver venous pressure
FMT, pilot study (NCT02469272)	MAFLD and MASH	Reduced liver steatosis
FMT vs. standard treatment (NCT02868164)	Liver cirrhosis due to MASH	Safety
FMT via duodenal infusion (NCT03803540)	MAFLD and MASH	Evaluated for effectiveness in MASH vs. MAFLD

Metformin, one of the most commonly used antidiabetic drugs, is known for its ability to modulate the gut microbiota by increasing the abundance of beneficial bacteria, particularly *Akkermansia muciniphila* and *Bifidobacterium* spp. (Wang et al., 2024; Halabitska et al., 2024b; Petakh et al., 2022). These bacteria enhance gut barrier integrity and reduce endotoxin-mediated inflammation (Di Vincenzo et al., 2024; Petakh et al., 2023b; Islam et al., 2023). Disruption of the intestinal barrier is a key mechanism contributing to the progression of MAFLD, particularly the development of liver fibrosis through the translocation of microbial metabolites and PAMPs into the portal circulation (An et al., 2022; Plaza-Díaz et al., 2020; Halabitska et al., 2024a). In addition to improving glycemic control, metformin enhances SCFA production, which improves insulin sensitivity and lipid metabolism (Petakh et al., 2023c; He, 2020; Mueller et al., 2021). Moreover, metformin influences bile acid metabolism by modulating the FXR, leading to reduced hepatic fat accumulation (Sun et al., 2018; Petakh et al., 2023a; Halabitska et al., 2024d). It also lowers TMAO levels, which are associated with cardiovascular risk in comorbidity (Jing et al., 2022; Dzhuryak et al., 2020; Belenichev et al., 2024). Collectively, these effects suggest a potential hepatoprotective role for metformin in MAFLD management, making it a promising therapeutic option (Kamyshnyi et al., 2021; Petakh et al., 2023d; Beygi et al., 2024).

Statins, including atorvastatin, simvastatin, rosuvastatin, pravastatin, and lovastatin, exhibit effects beyond lipid-lowering properties by modulating gut microbiota composition (Zivkovic et al., 2023; Morofuji et al., 2022; Repchuk et al., 2021). Studies have shown that statins reduce the abundance of pathogenic Gram-negative bacteria that produce LPS, potent triggers of inflammatory responses (McFarland et al., 2017; Bilyi et al., 2015; Luo et al., 2024). This is particularly relevant for MAFLD patients, as chronic inflammation drives disease progression from steatosis to steatohepatitis and fibrosis (Buchynskyi et al., 2024a; Gallego-Durán et al., 2022). Additionally, statins may enhance SCFA production, positively impacting glucose homeostasis, lipid metabolism, and insulin sensitivity (Sun et al., 2022; Halabitska et al., 2024c).

Angiotensin-converting enzyme (ACE) inhibitors, such as enalapril, lisinopril, ramipril, perindopril, and captopril, also influence gut microbiota by reducing inflammatory cytokine levels and modulating bacterial composition (Jaworska et al., 2021; Zheng et al., 2022). Some studies suggest these drugs increase the abundance of beneficial SCFA-producing bacteria while decreasing pro-inflammatory microorganisms and improving hepatic metabolic function in MAFLD patients (Pasta et al., 2025; Buchynskyi et al., 2023b).

Bile acid-modulating agents, including cholic acid, obeticholic acid, cholestyramine, and colesevelam hydrochloride, play a key role in gut microbiota–liver interactions (Chiang, 2013; Bertolini et al., 2022). Obeticholic acid, an FXR agonist, regulates bile acid metabolism and reduces endotoxemia by altering gut bacterial composition (Zhang et al., 2019; Li Y. et al., 2024). Dysregulation of bile acid homeostasis is linked to hepatic inflammation, highlighting the potential of FXR agonists in MAFLD treatment (Radun and Trauner, 2021; Belka et al., 2024).

As previously discussed, FXR signaling along the gut-liver axis influences BA synthesis and enterohepatic circulation, intestinal barrier integrity, and bacterial translocation, as well as modulating the gut microbiota, all of which are implicated in the pathogenesis of MASH. During MASH progression, a reduction in gut microbial diversity is observed, accompanied by an increase in the relative abundance of *Streptococcus* and Gram-negative bacteria (Henry et al., 2022).

Dysbiotic microbiota contribute to the disruption of the intestinal barrier, impacting the mucus lining, intercellular junctions within the epithelial layer, and the recently characterized gut vascular barrier (GVB) (Mouries et al., 2019). Evidence from animal models indicates that microbiota-mediated disruption of the GVB constitutes a critical antecedent to the development of MASH. Notably, FXR activation has been demonstrated to both prevent and ameliorate GVB disruption induced by dysbiosis, resulting in a reduction of bacterial translocation to the liver and a subsequent attenuation of inflammatory mediators (Mouries et al., 2019). Alterations in BAs composition, mediated by FXR activation both directly and through the fibroblast growth factor 15/19 pathway, have been shown to restore the diminished intestinal microbiota diversity associated with a high-fat diet, thereby contributing to the therapeutic management of MASH (Zhang et al., 2019). Furthermore, FXR activation has been shown to augment ileal expression of the antimicrobial peptides angiogenin-1 and alpha-5-defensin, both of which exhibit diminished expression in cirrhotic rat models (Úbeda et al., 2016).

Emerging therapeutic agents, such as maralixibat (an ASBT inhibitor), cilofexor, tropifexor, and nidufexor (FXR agonists), have demonstrated significant effects on the gut-liver axis (Almeqdadi and Gordon, 2024; Duan et al., 2022). These drugs modulate gut microbiota by regulating bile acid metabolism, which can mitigate inflammatory processes and improve liver function in MAFLD patients (Guo et al., 2023; Zherebiatiev and Kamyshnyi, 2016). ASBT inhibition, for instance, decreases bile acid reabsorption in the ileum, altering their availability to gut bacteria and shifting the microbial composition toward a less inflammatory profile (Fleishman and Kumar, 2024; Out et al., 2015).

Overall, evidence suggests that pharmacological agents used to treat metabolic and cardiovascular diseases can directly or indirectly impact gut microbiota, which is crucial for MAFLD development and progression (Gupta et al., 2022; Halabitska et al., 2024e; Drożdż et al., 2021).

## 8 Gut microbiota, MAFLD and nutrition

Over the past few years, substantial evidence has emerged elucidating the complex interplay between dietary patterns and the intestinal microbiota (Shortt et al., 2018; Mokkala et al., 2020). Notably, short-term alterations in gut microbial composition have been observed following adherence to a low-carbohydrate diet (LCD) (Henao-Mejia et al., 2012) and a ketogenic diet (KD) (Ang et al., 2020). Notably, substantial differences in the relative abundances of Firmicutes, Bifidobacterium, Bacteroidetes and Actinobacteria have been identified across ketogenic diet (KD), low-fat diet (LFD), and HFD, with Bifidobacterium exhibiting the most pronounced decline following KD (Ang et al., 2020). Moreover, a negative correlation has been observed between Bifidobacterium abundance and β-hydroxybutyrate (β-OHB) concentration in the intestinal lumen, suggesting that β-OHB inhibits Bifidobacterium growth—a relationship that has been further validated through *in vitro* studies (Ang et al., 2020). Additionally, the microbiota signature associated with KD has been linked to a reduction in intestinal pro-inflammatory Th17 cells (Ang et al., 2020).

Differential formulations of high-fructose diets have been shown to elicit distinct perturbations in gut microbiota composition. Specifically, high-fructose corn syrup (HFCS) consumption resulted in a reduction of butyrate-producing bacteria and a decrease in the *Firmicutes*/*Bacteroidetes* ratio, whereas a high-fructose diet derived from fruits induced an opposing shift (Beisner et al., 2020). This observation is clinically significant, as an elevated *Firmicutes*/*Bacteroidetes* ratio has been implicated in the pathogenesis of metabolic syndrome (Horne et al., 2020; Magne et al., 2020). Furthermore, individuals exhibiting a higher relative abundance of *Akkermansia muciniphila* demonstrated enhanced improvements in insulin sensitivity markers and other clinical parameters following calorie restriction interventions (Dao et al., 2016). Notably, both low-carbohydrate diets (LCDs) and ketogenic diets (KDs) have been shown to increase the abundance of *Akkermansia muciniphila* (Olson et al., 2018). Moreover, oral supplementation with *Akkermansia muciniphila* resulted in improved insulin sensitivity and cholesterol levels in overweight/obese, insulin-resistant human subjects (Depommier et al., 2019).

## 9 Host genetics and gut microbiota in MAFLD patients

The roles of both genetic factors and the microbiome in MAFLD have been extensively investigated. In a comparative analysis involving 44 obese adolescents with MAFLD and 29 obese adolescents without MAFLD, the abundance of fecal *Gemmiger* and *Oscillospira*, alongside the presence of the PNPLA3 rs738409 variant, demonstrated predictive value for hepatic fat fraction (Monga Kravetz et al., 2020).

In a separate investigation involving 10 patients with simple steatosis and 22 patients with steatohepatitis, a significant decrease in the abundance of *Desulfobacteraceae* bacteria was observed. Conversely, fungal genera including *Fusarium*, *Candida*, *Aspergillus*, and *Saccharomyces* exhibited increased abundance in patients harboring the PNPLA3 rs738409 GG genotype (Mascardi et al., 2023).

A microbiome analysis performed on liver tissue samples from 116 patients with MAFLD, comprising 19 controls, 44 patients with metabolic dysfunction-associated steatotic liver (MASL), and 53 patients with MASH, substantiated the association between host genetics and the hepatic microbiome. Specifically, individuals carrying the PNPLA3 rs738409 G allele exhibited an enrichment of Enterobacter and Marivota in liver tissue, whereas carriers of the TM6SF2 rs58542926 T allele showed an increased abundance of *Pseudoalteromonas* and Megamonas.

Carriers of the MBOAT7 rs641738 T allele exhibited a depletion of *Butyricicoccus* and *Streptococcus*, whereas carriers of the HSD17B13 rs72613567 TA allele demonstrated decreased abundances of *Fusobacterium* and *Parasutterella* (Mancabelli et al., 2017). Notably, the strongest associations observed were between *Enterobacter* and the PNPLA3 rs738409 polymorphism, and between *Pseudoalteromonas* and the TM6SF2 rs58542926 polymorphism. These two genera belong to the Gamma proteobacteria class, which has been associated with more severe forms of MAFLD (Sookoian et al., 2020).

In addition to the influence of host genetic variants, MAFLD-related gene expression profiles also impact the composition of the gut microbiome. G protein-coupled receptor 35 (GPR35), an orphan receptor highly expressed in gut epithelial and myeloid cells, has been shown to mitigate obesity-related MASH through the regulation of hepatic cholesterol homeostasis (Wei et al., 2023).

Polymorphisms within the GPR35 gene have been associated with intestinal inflammation, metabolic stress, and T2DM (Ellinghaus et al., 2013). Global and intestinal-specific deletions of GPR35 have been demonstrated to induce gut dysbiosis and increase susceptibility to hepatic steatosis and metabolic syndrome. Further research has revealed that the absence of GPR35 results in an increased abundance of *Ruminococcus gnavus* in the gut. This increase, in conjunction with a high-fat diet, disrupts lipid metabolism and promotes hepatic fat accumulation through the production of indoxylsulfuric acid, a uraemic toxin.

## 10 Gut mycobiome and virome in MAFLD pathogenesis

Emerging evidence indicates that gut fungi (the mycobiome) contribute to MAFLD progression. Clinical studies have identified shifts in fungal composition in patients with MAFLD, with an overrepresentation of opportunistic yeasts such as *Candida albicans* and certain molds (*Mucor* spp.) especially in those with NASH or advanced fibrosis (Hartmann and Schnabl, 2023; Zeng and Schnabl, 2024). For example, fecal *C. albicans* and *Mucor* levels are significantly higher in NASH and fibrotic NAFLD patients compared to those with simple steatosis (Hartmann and Schnabl, 2023). These fungal taxa correlate with clinical indices of liver injury and dyslipidemia.

Mechanistic links between the mycobiome and hepatic inflammation are beginning to be uncovered. Overgrowth of *C. albicans* in the gut may drive liver disease via translocation of fungal products. Patients with NAFLD show elevated anti-Candida antibody titers that correlate with disease activity, implying that fungal antigens breach the gut barrier and stimulate hepatic immune responses (Demir et al., 2022; Hartmann et al., 2021).

Fungal cell wall components like β-glucans can activate pattern recognition receptors in the liver: in particular, β-glucans engage the C-type lectin receptor Dectin-1 on Kupffer cells and macrophages, triggering NF-κB signaling and NLRP3 inflammasome activation (Wang et al., 2023; Li L. et al., 2024). In a recent study, Dectin-1 was upregulated in the livers of HFD-induced NAFLD mice and human NASH patients; genetic knockout or pharmacologic blockade of Dectin-1 markedly attenuated steatosis, inflammation and fibrosis in NAFLD models (Wang et al., 2023). These findings suggest that gut fungi (through β-glucans and other molecules) exacerbate hepatic macrophage activation and inflammatory injury (Hartmann and Schnabl, 2023). Likewise, *C. albicans* produces a pore-forming toxin, candidalysin, which has been shown to worsen alcoholic liver disease and is hypothesized to similarly promote NAFLD/NASH pathogenesis (Li L. et al., 2024). Conversely, not all fungi are deleterious—certain commensal yeasts may have protective effects. For instance, Saccharomyces species produce immunomodulatory β-glucans that can skew immune responses toward anti-inflammatory pathways and improve metabolic parameters in experimental models (Horneck Johnston et al., 2024; Brown and Gordon, 2001).

Beyond bacteria and fungi, the gut virome (community of viruses, including bacteriophages and eukaryotic viruses) has gained recognition as a key player in NAFLD/MAFLD. Viruses are abundant in the intestine and intimately interact with bacterial and mammalian hosts, yet their role in liver disease has only recently been explored (Carding et al., 2017; Lang et al., 2020). In patients with NAFLD, especially those with more advanced disease, significant alterations in the fecal virome have been observed. Notably, a 2021 metagenomic study demonstrated that individuals with severe NASH or fibrosis had a reduction in overall intestinal viral diversity, particularly a loss of gut bacteriophage richness, compared to those with milder disease or healthy controls (Lang et al., 2020). The proportion of bacteriophages within the total viral community was markedly lower in advanced NAFLD, suggesting a relative expansion of eukaryotic viruses or prophages in these patients (Lang et al., 2020). These findings raise the possibility that changes in the virome could serve as biomarkers of disease severity and might actively influence disease progression.

Mechanistically, bacteriophages (phages) shape the gut ecosystem by regulating bacterial population dynamics and gene content (Liang and Bushman, 2021). Lytic phages can directly affect the gut-liver axis by lysing Gram-negative bacteria in the intestine, which leads to the release of pro-inflammatory bacterial components such as lipopolysaccharide (LPS) into the gut lumen (Hsu et al., 2021). Beyond phages, the gut virome includes eukaryotic viruses (viruses infecting human cells, fungi, or protozoa, as well as dietary plant viruses), though their contributions to MAFLD are still poorly characterized (Mirzaei and Maurice, 2017).

## 11 Conclusion

MAFLD is a complex metabolic disorder characterized by hepatic lipid accumulation and subsequent inflammation, closely linked to metabolic syndrome and obesity. The pathogenesis of MAFLD involves multiple factors, including insulin resistance, lipotoxicity, oxidative stress, and inflammatory responses. The gut microbiota plays a crucial role in MAFLD development, with dysbiosis contributing to liver inflammation through various mechanisms.

This review has highlighted the intricate relationship between the gut microbiota and MAFLD, emphasizing the importance of understanding the complex interplay between the gut microbiome, intestinal barrier integrity, and hepatic immune responses. The identification of specific gut microbiome signatures in MAFLD patients offers potential diagnostic and therapeutic targets, while the influence of microbial metabolites on hepatic function and immune responses underscores the need for further research.

The findings of this review have significant implications for the prevention and treatment of MAFLD. Modulation of the gut microbiota through probiotics, prebiotics, or fecal microbiota transplantation may offer a novel therapeutic approach for MAFLD management. Additionally, the development of diagnostic tools based on gut microbiome signatures may enable early detection and monitoring of MAFLD progression.

In conclusion, the relationship between the gut microbiota and MAFLD is complex and multifaceted, involving various mechanisms and pathways. Further research is necessary to elucidate the precise mechanisms underlying this relationship and to develop effective therapeutic strategies for the prevention and treatment of MAFLD.
